# Epstein-Barr Virus-Positive Mucocutaneous Ulcer With Medication-Related Osteonecrosis of the Jaw Arising in the Mandible of a Rheumatoid Arthritis Patient: A Case Report

**DOI:** 10.7759/cureus.61280

**Published:** 2024-05-28

**Authors:** Naomi I Kanno, Takashi Ito, Shohei Takaoka, Kenji Yamagata, Hiroki Bukawa

**Affiliations:** 1 Oral and Maxillofacial Surgery, University of Tsukuba, Institute of Medicine, Ibaraki, JPN

**Keywords:** rheumatoid arthritis, lpd, lymphoproliferative disorder, ebvmcu, ebv-positive mucocutaneous ulcer, mronj, medication-related osteonecrosis of the jaw, mtx, methotrexate

## Abstract

This study presents a rare case of an Epstein-Barr virus-positive mucocutaneous ulcer (EBVMCU) co-existing with medication-related osteonecrosis of the jaw (MRONJ) in the mandible of a 54-year-old Japanese man who complained of painful swelling of the left mandibular gingiva over the past three months. The patient had a history of methotrexate (MTX) and bisphosphonates (BPs) use. Intraoral examination revealed a 35 mm large ulcerative lesion with marginal gingival swelling and bone exposure on the left side of the mandible. A biopsy was performed, confirming the diagnosis of EBVMCU with MRONJ. Due to the enlargement of the bone exposure, marginal resection of the mandible was performed under general anesthesia as a treatment for residual MRONJ. At the two-year follow-up, no evidence of recurrence was observed.

## Introduction

Epstein-Barr virus (EBV), also known as human herpes virus 4 (HHV-4), mainly infects B cells (B lymphocytes) via saliva. Diseases caused by EBV include lymphoproliferative diseases (LPD), infectious mononucleosis, cancers of the mid-pharynx and stomach, and many others [1]. EBV-positive lymphoproliferative disorder (EBV-positive LPD) is a disease in which EBV reactivation in an immunosuppressed state causes the proliferation of tumorigenic B cells. EBV-positive mucocutaneous ulcers (EBVMCU) were first reported in 2010 [2] and were classified as a new disease concept distinct from immunodeficiency-related lymphoproliferative disorders in tumors of hematopoietic and lymphoid tissues by the fourth revision of the WHO classification in 2017. The third edition of the WHO classification in 2001 initially classified this condition as "MTX-associated lymphoproliferative diseases (MTX-LPD)." However, in the fourth edition in 2008, it was classified as "other iatrogenic immunodeficiency-associated lymphoproliferative diseases." Subsequently, in the revised edition in 2017 (4th edition), it was classified as "other iatrogenic immunodeficiency-associated lymphoproliferative disorders" including drugs other than methotrexate (MTX). Furthermore, it was classified as EBVMCU as a disease caused by various immunodeficiency conditions (i.e., medical immunosuppression, aging, HIV infection, and immunocompromised primary immunity).

Medication-related osteonecrosis of the jaw (MRONJ) is a serious adverse event that occurs in patients with cancer or osteoporosis previously treated with strong antiresorptive or angiogenesis inhibitors, including bisphosphonates (BPs; e.g., zoledronic acid, alendronate) and anti-RANKL monoclonal antibodies (e.g., denosumab). It is used to manage bone metastases in cancer patients and to prevent fragility fractures in osteoporosis patients [3]. MTX, which has immunosuppressive properties, is the first-line treatment for rheumatoid arthritis (RA). Additionally, patients with RA have a strong association with osteoporosis due to the disease itself, steroid use, and the use of BPs and anti-RANKL monoclonal antibodies.

This study aims to report a case of an RA patient taking MTX and BPs diagnosed with MRONJ and EBVMCU in the mandible, which required spontaneous remission of EBVMCU by MTX withdrawal and jaw osteotomy for MRNOJ.

## Case presentation

A 54-year-old man was referred to the Department of Oral and Maxillofacial Surgery, University of Tsukuba Hospital, with complaints of swelling and pain in the left mandibular gingiva. He had a history of RA (Stage IV, 2008) and osteoporosis. His medication history included MTX 16 mg/week (2008-; > 13 years), prednisolone (PSL) 5 mg/day, and ibandronic acid (BonvivaR) (2017-; > 3 years).

**Figure 1:**
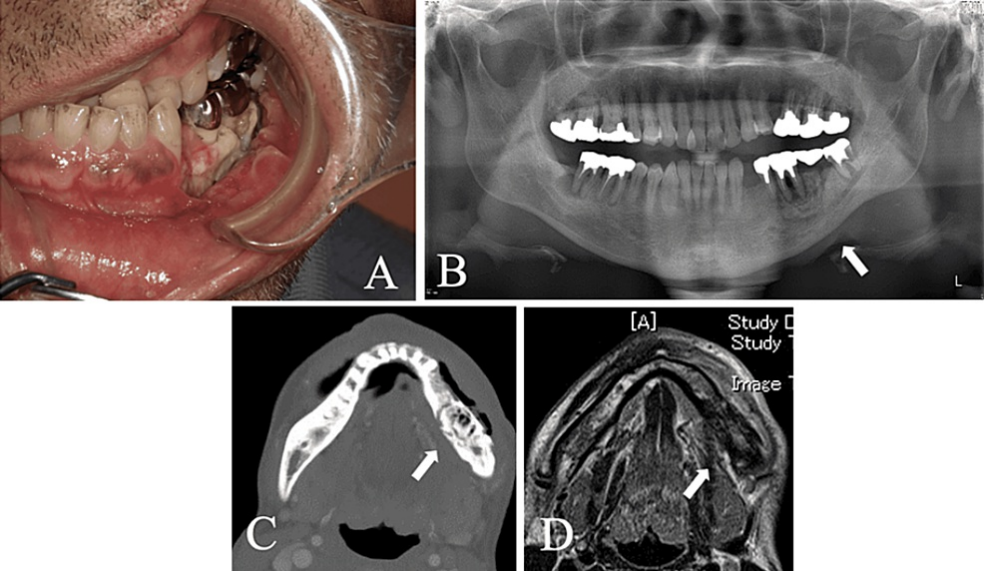
Image findings at initial examination. A: Intraoral examination; A 35mm-sized ulcerative lesion with marginal swelling, bone exposure, and drainage is seen on the lower left 5-8 gingiva. B: Panoramic X-ray findings; rotund-like bone resorption and bone separation in the left lower sixth. C: Contrast-enhanced CT findings (horizontal section); rotting bone-like bone resorption and bone separation in the left mandibular molar region. D: Contrast-enhanced MR findings; T2-weighted image shows abnormal marrow signal in the left mandibular body and uniform thickness of swelling in the buccal gingival region.

He was referred to the Department of Oral Surgery of a nearby hospital, where he was diagnosed with MRONJ and followed up conservatively with ibandronate withdrawal three months prior, but his mandibular condition did not improve, and he was referred to our department. Intraoral examination revealed a 35 mm large ulcerative lesion with marginal swelling in the lower left molar gingiva. No indurations were observed around the lesions. Bone exposure and drainage were observed in the same area (Figure 1A). Extraoral examination revealed an asymmetrical facial appearance, with swelling in the left submandibular region and paresthesia in the inferior alveolar nerve area. Panoramic radiography revealed moderate horizontal bone resorption throughout the jaw, and the left mandibular molar showed evidence of decay-like bone resorption and separation (Figure 1B). Contrast-enhanced CT revealed destruction and decay during bone formation in the left mandibular molar area (Figure 1C). Contrast-enhanced MR images revealed a T2-weighted image showing an abnormal bone marrow signal in the left mandibular body and uniform thickness of swelling in the buccal gingival region (Figure 1D). 

**Figure 2:**
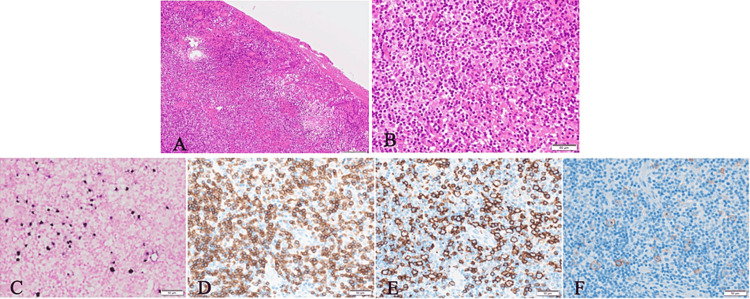
Pathological and immunohistological findings (at biopsy). A: Hematoxylin and eosin (HE) staining, ×100; erosions and severe inflammatory cell infiltration. B: HE staining, ×400; large lymphocytes present sporadically. C: EBV ISH (Epstein-Barr virus in situ hybridization), ×400; positive for large lymphocytes. D: CD3, ×400; positive for small T cells. E: CD20, ×400; positive for small to large B cells. F: CD30, ×400; positive for large lymphocytes.

The clinical imaging diagnosis was suspected of both EBVMCU (MTX-related lymphoproliferative disease) and MRONJ. Biopsy of the gingiva and extraction of the left lower first molar were performed under local infiltration anesthesia. Suspecting the possibility of drug-induced ulceration due to MTX, MTX was withdrawn after the day of the biopsy. Histopathological findings of the gingival biopsy specimens revealed a highly acute to chronic cellular infiltrate, multilayered squamous epithelium with granulation tissue hyperplasia, small lymphocytes, and a few large lymphocytes with blister-like nuclear chromatin (Figures 2A, 2B). Immunohistochemical findings of the biopsy specimens were positive for CD3, CD10, CD20, Pax-5, CD30, BCL-6, MUM-1, and MIB-1, and EBV staining was positive for EBV (Figures 2C-2F).

**Figure 3:**
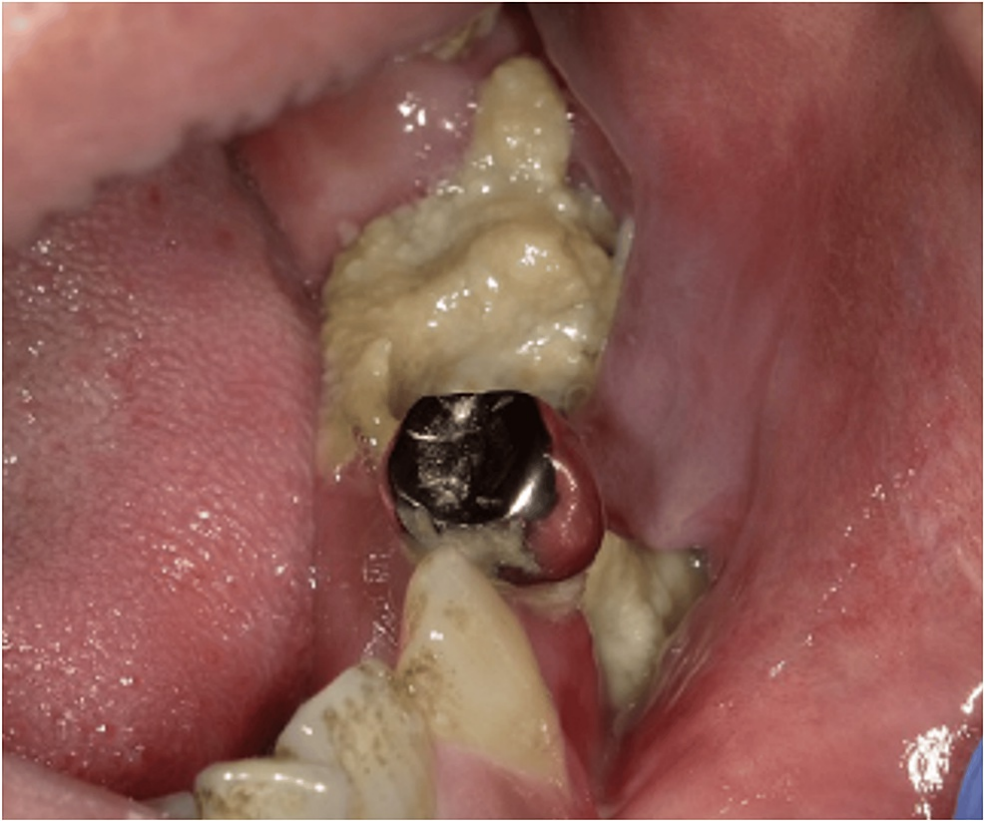
Image findings after four months of MTX withdrawal. MTX: Methotrexate.

After four weeks of MTX withdrawal, gingival erythema, and swelling had almost completely resolved; the disappearance of ulceration of both the gingiva and tongue was observed. Exposure of decayed bone due to MRONJ gradually increased, and pus drainage persisted. After four months of MTX withdrawal, redness and swelling of the gingiva remained absent, and the EBVMCU was diagnosed as already in remission (Figure 3).

**Figure 4:**
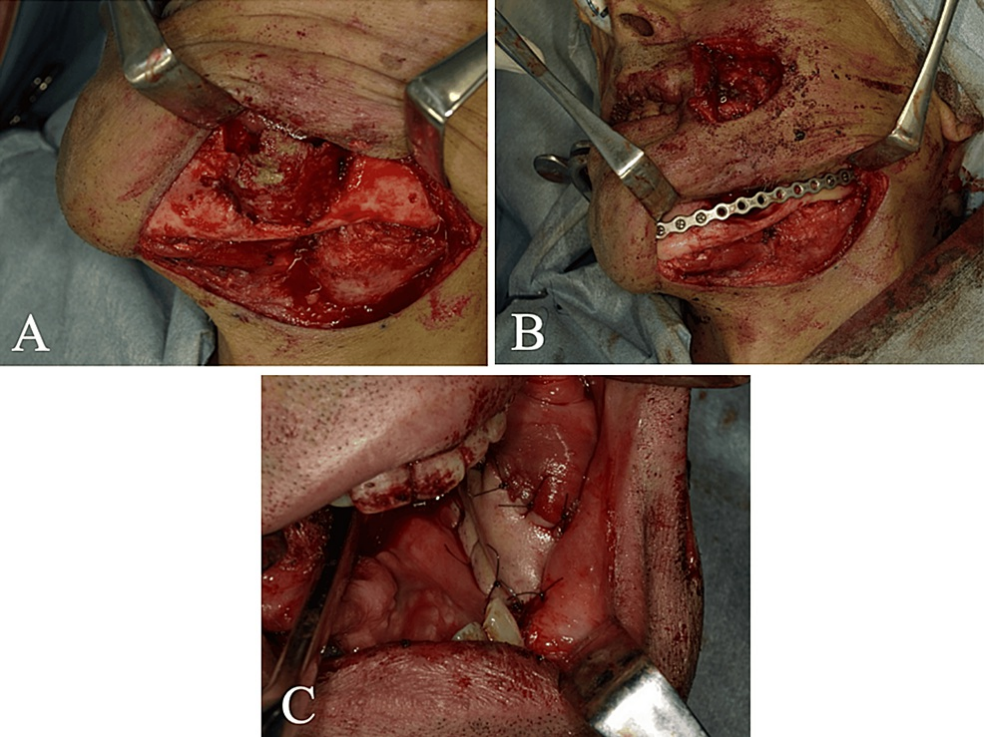
Intraoperative findings. A: Before necrotic bone removal. B: After removal of decayed bone and fixation of titanium plate. C: After reconstruction with nasolabial flap.

After infection control by medication with amoxicillin, marginal resection of the necrotic bone of the mandible was performed under general anesthesia to treat the MRONJ (Figure 4A). The remaining mandibular submargin bone was thin, so it was reinforced with a titanium plate (Figure 4B). In addition, immediate soft-tissue reconstruction with a pedicled nasolabial flap was performed because of the large gingival defect caused by the EBVMCU and MRONJ (Figure 4C). 

**Figure 5:**
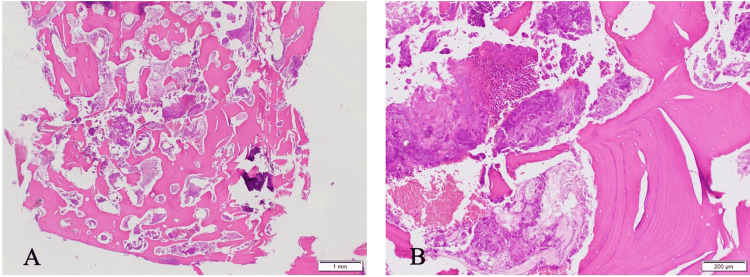
Pathological findings (at surgery). A: Hematoxylin and eosin (HE) staining, ×20; necrotic bone, osteoblasts were absent, and the surface of the bone trabeculae presented with a worm-eaten appearance. B: HE staining, ×100; osteoblast-displaced necrotic bone, actinomycetes.

The histopathological analysis of the surgically removed specimens showed necrotic bone, osteoblasts were absent, and the surface of the bone trabeculae presented with a worm-eaten appearance (Figure 5A). Actinomycotic colonies were found on the bone trabeculae (Figure 5B).

**Figure 6:**
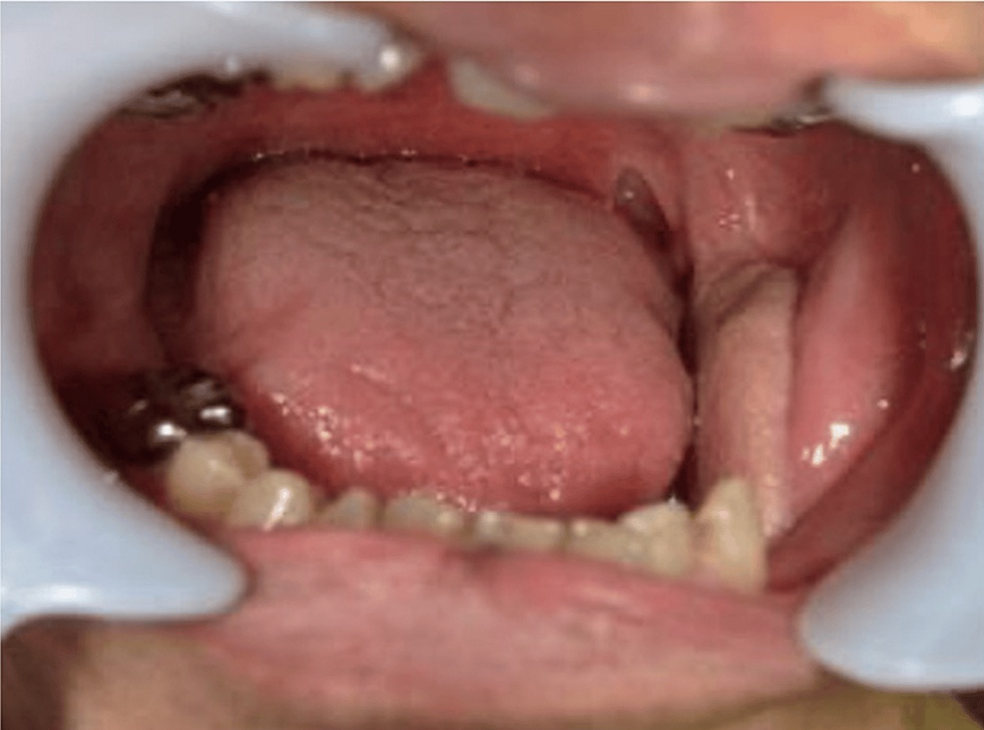
Image findings two years after the surgery.

There were no changes in osteosclerosis. The final histopathological diagnosis was EBVMCU with MRONJ of the jaw. Two years after the surgery, the patient was doing well without recurrence (Figure 6). His RA was well-controlled with PSL 2 mg/day, iguratimod (IGU) 50 mg/day, and bucillamine (BUC) 200 mg/day, without MTX.

## Discussion

MTX-LPD was first described by Ellman et al. in 1991 as lymphoma occurring in patients with rheumatoid arthritis receiving MTX [4]. In the 2008 WHO classification, MTX-LPD was defined as a representative disease of immunodeficiency-associated lymphoproliferative disorders, which are autoimmune diseases such as RA. This definition excluded immunodeficiency-associated lymphoproliferative disorders, and MTX-associated lymphoproliferative disorders (MTX-LPDs), which are lymphoproliferative disorders that occur in patients receiving immunosuppressive therapy for autoimmune diseases, such as RA and Crohn's disease; it excluded immunodeficiency-associated lymphoproliferative disorders. MTX is an anti-rheumatic drug widely used as a first-line treatment for autoimmune diseases, especially RA, and has an excellent inhibitory effect on joint destruction. However, its side effects include gastrointestinal symptoms (stomatitis, nausea, diarrhea, etc.), liver damage, infection, myelosuppression, interstitial pneumonia, and LPD.

Conversely, EBVMCU was first reported in 2010 and does not belong to any of the four categories of immunodeficiency-related LPD according to the WHO classification of diseases (LPD associated with primary immunodeficiency, lymphoma associated with HIV infection, post-transplant LPD, and other iatrogenic immunodeficiency-related LPD) and was a newly classified disease in the 2017 revision of the fourth edition of the WHO classification [2,5]. Prevalent sites are the oral mucosa, gastrointestinal tract, and skin, with more than 70% of cases reported to occur on the oral mucosa [6]. Lymphoproliferative disease presents as relatively shallow, localized, irregularly shaped ulcers with well-defined borders and no generalized involvement [2]. It is caused by various immunodeficiency conditions (e.g., medical immunosuppression, aging, HIV infection, and primary immunodeficiency) [7-12] and usually goes into spontaneous or complete remission with an improvement in the immunosuppressive state. Medically-induced immunosuppression is caused by immunosuppressive drugs such as azathioprine (AZA) and cyclosporine (CyA), in addition to MTX. There is a report of the discovery that EBV-infected B cells are distributed in large numbers in the tonsillar rings of Waldeyer's and that the oral mucosa is susceptible to microscopic damage by mechanical stimuli [2]. There is also a report on the relationship between periodontal disease and oral hygiene [13].

The WHO classification of the disease has evolved over different reporting periods, designating some cases as MTX-LPD and others as EBVMCU. Fifteen Japanese cases of lymphoproliferative oral mucosal disease associated with osteonecrosis of the jaw, including the present case, were reviewed (Table 1). Although several cases, each of EBVMCU or MRONJ have been reported, non-Japanese cases of complications EBVMCU with MRONJ were not reported to the best of our knowledge. Twelve of 15 patients were elderly patients aged 65 years or older, and the incidence was higher among females (six males and nine females). The most common site of onset is the gingiva, with ulceration and pain as the main symptoms. The primary disease was RA in 14 patients and arthritis in one patient, all of whom were taking MTX. Fourteen patients were followed up after MTX withdrawal and one patient was followed up after MTX dose reduction; all patients had a spontaneous remission. Additionally, surgical treatment for MRONJ, such as sequestrectomy and jaw osteotomy, or spontaneous separation of decayed bone after remission of EBVMCU, improved MRONJ, and all patients had a good course without recurrence.

**Table 1:** Reported cases of EBVMCU (or MTX-LPD) with MRONJ. EBVMCU: Epstein-Barr virus-positive mucocutaneous ulcer, MTX-LPD: Methotrexate-associated lymphoproliferative disorders, MRONJ: Medication-related osteonecrosis of the jaw, BP: Bisphosphonate, M: Male, F: Female, RA: Rheumatoid arthritis, JIA: Juvenile idiopathic arthritis, OP: Osteoporosis, WDL: Withdrawal, NM: No medication.

	Year	Age	Gender	Sites affected	Primary symptoms	Previous diseases	MTX (Duration of medications; year)	BP (Duration of medications; year)	MTX (Medication after disease onset)	BP (Medication after disease onset)	Duration of EBVMCU remission; week	Recurrence of EBVMCU	Treatment of the MRONJ
1	2012	70	M	Upper and lower molar gingiva	Pain	RA	0.5	0	Dose reduction	NM	4	−	Spontaneous separation of necrotic bone
2	2012	66	F	Upper molar gingiva	Exposed bone	RA OP	12	3.8	WDL	WDL	12	−	Surgical debridement
3	2012	64	F	Upper and lower anterior gingiva, Lower molar gingiva	Pain	RA	3	2	WDL	WDL	4	−	Surgical debridement
4	2012	40	F	Upper anterior gingiva, Palate	Pain, Swelling	JIA	4.9	0	WDL	NM	12	−	Spontaneous separation of necrotic bone
5	2013	84	F	Lower anterior gingiva	Pain, Swelling	RA	1.6	0	WDL	NM	4	−	Spontaneous separation of necrotic bone
6	2014	74	F	Upper anterior gingiva	Exposed bone	RA	10	0	WDL	NM	7	−	Surgical debridement
7	2015	80	M	Upper molar gingiva	Pain, Ulcer	RA	6.3	0	WDL	NM	40	−	Spontaneous separation of necrotic bone
8	2015	87	M	Upper anterior gingiva	Ulcer	RA OP	12	5	WDL	WDL	8	−	Spontaneous separation of necrotic bone
9	2016	82	M	Lower molar gingiva	Pain	RA	7.4	0	WDL	NM	5	−	Spontaneous separation of necrotic bone
10	2017	70	F	Upper and lower molar gingiva	Exposed bone	RA	15	0	WDL	NM	5	−	Resection
11	2017	72	F	Upper anterior gingiva	Pain, Exposed bone	RA OP	7.3	0.7	WDL	WDL	4	−	Surgical debridement
12	2018	81	F	Upper molar gingiva	Pain	RA	4	7	WDL	Unknown	2	−	Surgical debridement
13	2018	72	M	Upper molar gingiva	Pain	RA	7	0	WDL	NM	2	−	No treatment
14	2022	77	F	Upper anterior gingiva	Pain, Bleeding	RA OP	7.2	Unknown	WDL	Unknown	3	−	Surgical debridement
15	2022	54	M	Lower molar gingiva	Pain, Swelling, Exposed bone	RA OP	12	3.3	WDL	WDL	4	−	Surgical debridement

The risk of osteoporosis is 1.3 times higher in patients with RA and 1.7 times higher in those with proximal femoral fractures due to RA. This risk increases further when the effects of long-term steroid therapy are considered. Therefore, the use of BPs and anti-RANKL antibodies, which are known to improve bone density, is recommended. In recent years, pharmacotherapy for RA has evolved, with an increasing number of biological products being used in combination with other drugs to enhance therapeutic efficacy; however, this is expected to become more complex due to the aging of the population and the combination of various drugs. 

## Conclusions

This report presented the rare case of a rheumatoid arthritis patient taking methotrexate (MTX) and bisphosphonate diagnosed with medication-related osteonecrosis of the jaw (MRONJ) and Epstein-Barr virus-positive mucocutaneous ulcer (EBVMCU) in the mandible, which required spontaneous remission of EBVMCU by MTX withdrawal and jaw osteotomy for MRNOJ. The patient had two different drug-related conditions that caused complications in the oral cavity. One limitation of this study is the small number of current reports, and it is important to conduct ongoing studies accumulating more cases.

## References

[1] Dunmire SK, Verghese PS, Balfour HH, Jr Jr (2018). Primary Epstein-Barr virus infection. J Clin Virol.

[2] Dojcinov SD, Venkataraman G, Raffeld M, Pittaluga S, Jaffe ES (2010). EBV positive mucocutaneous ulcer--a study of 26 cases associated with various sources of immunosuppression. Am J Surg Pathol.

[3] Aguirre JI, Castillo EJ, Kimmel DB (2021). Preclinical models of medication-related osteonecrosis of the jaw (MRONJ). Bone.

[4] Ellman MH, Hurwitz H, Thomas C, Kozloff M (1991). Lymphoma developing in a patient with rheumatoid arthritis taking low dose weekly methotrexate. J Rheumatol.

[5] Swerdlow SH, Campo E, Harris NL, Jaffe ES, Pileri SA, Stein H, Thiele J (2017). WHO classification of tumours of haematopoietic and lymphoid tissues. https://publications.iarc.fr/Book-And-Report-Series/Who-Classification-Of-Tumours/WHO-Classification-Of-Tumours-Of-Haematopoietic-And-Lymphoid-Tissues-2017.

[6] Ikeda T, Gion Y, Yoshino T, Sato Y (2019). A review of EBV-positive mucocutaneous ulcers focusing on clinical and pathological aspects. J Clin Exp Hematop.

[7] Ikeda T, Gion Y, Sakamoto M (2020). Clinicopathological analysis of 34 Japanese patients with EBV-positive mucocutaneous ulcer. Mod Pathol.

[8] Satou A, Banno S, Hanamura I (2019). EBV-positive mucocutaneous ulcer arising in rheumatoid arthritis patients treated with methotrexate: Single center series of nine cases. Pathol Int.

[9] Hart M, Thakral B, Yohe S (2014). EBV-positive mucocutaneous ulcer in organ transplant recipients: a localized indolent posttransplant lymphoproliferative disorder. Am J Surg Pathol.

[10] Fukuzawa S, Yamagata K, Terada K, Uchida F, Ishibashi-Kanno N, Bukawa H (2022). Age related immunosenescence Epstein-Barr virus-positive mucocutaneous ulcer of the palate mimicking medication-related osteonecrosis of the jaw. Indian J Otolaryngol Head Neck Surg.

[11] Daroontum T, Kohno K, Eladl AE (2018). Comparison of Epstein-Barr virus-positive mucocutaneous ulcer associated with treated lymphoma or methotrexate in Japan. Histopathology.

[12] Bunn B, van Heerden W (2015). EBV-positive mucocutaneous ulcer of the oral cavity associated with HIV/AIDS. Oral Surg Oral Med Oral Pathol Oral Radiol.

[13] Sato K, Takahashi N, Kato T (2017). Aggravation of collagen-induced arthritis by orally administered Porphyromonas gingivalis through modulation of the gut microbiota and gut immune system. Sci Rep.

